# H-Channels Affect Frequency, Power and Amplitude Fluctuations of Neuronal Network Oscillations

**DOI:** 10.3389/fncom.2015.00141

**Published:** 2015-11-19

**Authors:** Oscar J. Avella Gonzalez, Huibert D. Mansvelder, Jaap van Pelt, Arjen van Ooyen

**Affiliations:** Department of Integrative Neurophysiology, Center for Neurogenomics and Cognitive Research, VU University AmsterdamAmsterdam, Netherlands

**Keywords:** h-channels, oscillations, synchrony, amplitude fluctuations, computational model

## Abstract

Oscillations in network activity are ubiquitous in the brain and are involved in diverse cognitive functions. Oscillation characteristics, such as power, frequency, and temporal structure, depend on both network connectivity and intrinsic cellular properties, such as ion channel composition. An important class of channels, with key roles in regulating cell excitability, are h-channels. The h-current (I_h_) is a slow, hyperpolarization-activated, depolarizing current that contributes to neuronal resonance and membrane potential. The impact of I_h_ on network oscillations, however, remains poorly understood. To elucidate the network effects of I_h_, we used a computational model of a generic oscillatory neuronal network consisting of inhibitory and excitatory cells that were externally driven by excitatory action potentials and sustained depolarizing currents. We found that I_h_ increased the oscillation frequency and, in combination with external action potentials, representing input from areas outside the network, strongly decreased the synchrony of firing. As a consequence, the oscillation power and the duration of episodes during which the network exhibited high-amplitude oscillations were greatly reduced in the presence of I_h_. Our results suggest that modulation of I_h_ or impaired expression of h-channels, as observed in epilepsy, could, by affecting oscillation dynamics, markedly alter network-level activity and potentially influence oscillation-dependent cognitive processes such as learning, memory and attention.

## Introduction

Oscillations in electrical activity are a hallmark of many brain networks (Gray et al., 1989; Fisahn et al., 1998; Csicsvari et al., 2003; van Aerde et al., 2008) and are associated with various cognitive functions, such as attention (Fries et al., 2001; Dehaene and Changeux, 2005; Buia and Tiesinga, 2006), temporal binding (Gray et al., 1989; Engel et al., 1999, 2001), learning (Miltner et al., 1999; Caplan et al., 2001), working memory (Raffone and Wolters, 2001; Howard et al., 2003; Haenschel et al., 2009), and memory consolidation (Axmacher et al., 2006). Network oscillations, as measured in EEG and extracellular field recordings, are produced by the rhythmic and synchronized firing of large numbers of cells (Buzsaki and Draguhn, 2004; Börgers et al., 2005; Womelsdorf and Fries, 2007) and are thought to arise from interacting populations of excitatory and inhibitory neurons (Tiesinga et al., 2001; Börgers and Kopell, 2005; Börgers et al., 2005).

Interestingly, the amplitude (or power) of ongoing oscillations often fluctuates strongly, with high-amplitude episodes (HAEs) alternating erratically with low-amplitude episodes (LAEs; Poil et al., 2008, 2011; Montez et al., 2009; van Aerde et al., 2009; Freyer et al., 2011). Oscillation amplitude is proportional to the number of simultaneously firing cells (Reichinnek et al., 2010; Pettersen et al., 2012), and LAEs may reflect episodes in which fewer cells fire or in which they fire less synchronized. Amplitude fluctuations are observed in EEG recordings of the intact brain (Montez et al., 2009; Freyer et al., 2011) as well in extracellular field recordings of slice cultures (van Aerde et al., 2009; Poil et al., 2011), and are found in many brain areas, including prefrontal cortex (van Aerde et al., 2009) and hippocampus (Poil et al., 2011), and many frequency bands, ranging from theta (4–6 Hz) to gamma (25–80 Hz) (Kim et al., 2007; Linkenkaer-Hansen et al., 2007; Mann and Mody, 2009; van Aerde et al., 2009). Episodes with high oscillation amplitude may provide favorable conditions for synaptic plasticity (Avella Gonzalez et al., 2014), and memory-related tasks are often associated with sustained increases in oscillation amplitude (Palva et al., 2005; Montgomery and Buzsáki, 2007). Changes in the temporal pattern of amplitude fluctuations have been observed in Alzheimer's disease (Montez et al., 2006) and ADHD (Dockstader et al., 2008).

The origin of amplitude fluctuations in ongoing oscillations is poorly understood. Using network models of interconnected excitatory and inhibitory cells (Avella Gonzalez et al., 2012, 2014), we showed that amplitude fluctuations can arise from a temporary decrease in firing synchrony caused by the interference between network-generated oscillations and input originating from areas external to the network. The external input could come in the form of random spike trains (Avella Gonzalez et al., 2012) or in the form of oscillating activity from another network (Avella Gonzalez et al., 2014). The distributions of HAE and LAE durations in the model matched those observed in prefrontal cortex (van Aerde et al., 2008, 2009) and hippocampus (Poil et al., 2011). In the model, frequency and randomness of the external input (Avella Gonzalez et al., 2012) as well as network connectivity (Avella Gonzalez et al., 2014) influenced HAE and LAE duration.

In general, oscillations in brain networks depend on both network connectivity and intrinsic cellular properties, such as ion channel composition. An important class of channels, with key roles in regulating excitation in neural and cardiac tissues, are hyperpolarization-activated cation channels (h-channels; Chen et al., 2002; Biel et al., 2009; Kase and Imoto, 2012). The h-current (I_h_) is a depolarizing, non-inactivating, mixed Na^+^-K^+^ current (with a reversal potential of −30 mV) that activates slowly in response to hyperpolarization and deactivates slowly in response to depolarization. Because h-channels are partially open at rest, I_h_ induces a depolarizing shift in the resting membrane potential and decreases the resting membrane resistance. Furthermore, I_h_ acts as a high-pass filter, opposing slow changes in membrane potential. Together with the low-pass filtering due to the membrane time constant, I_h_ endows the cell with resonance, the property to respond selectively to inputs at a preferred frequency (Hutcheon and Yarom, 2000).

In the brain, I_h_ has important roles in controlling neuronal excitability (Pape and McCormick, 1989), dendritic integration (Magee, 1999), synaptic transmission and plasticity (Beaumont and Zucker, 2000; Nolan et al., 2004), motor learning (Nolan et al., 2003), and working memory (Wang et al., 2007). In addition, I_h_ may be involved in generating or regulating oscillatory brain activity, such as rhythmic pacemaker depolarization (McCormick and Bal, 1997; Dickson et al., 2000), sub-threshold oscillations in the entorhinal cortex (Dickson et al., 2000; Haas et al., 2007), thalamocortical oscillations (Steriade et al., 1993; Bal and McCormick, 1996), and oscillations in the hippocampus (Fisahn et al., 2002; Cunningham et al., 2003; Neymotin et al., 2013) and the prefrontal cortex (Vijayraghavan et al., 2007; Wang et al., 2007; Chu and Zhen, 2010). I_h_ has also been implicated in epilepsy, and modulation of I_h_ has been proposed as a potential attribute of novel anti-epileptic drugs (Chen et al., 2002). The unique biophysical properties and multifaceted aspects of I_h_, however, make it difficult to elucidate the network-level consequences of I_h_ and thus to predict the effect of I_h_ on brain oscillations.

Using a computational model of a generic neuronal network, we here studied the impact of I_h_ on network oscillations and in particular its impact on high- and low-amplitude episodes. In the model, cells were externally driven by sustained current input as well as trains of action potentials, and oscillations were generated by the interaction between excitatory and inhibitory cells (Tiesinga et al., 2001; Börgers and Kopell, 2005; Börgers et al., 2005). We found that I_h_ increased the oscillation frequency and, in combination with external action potential input, strongly decreased the synchrony of firing, as a result of which the oscillation power and the duration of episodes with high-amplitude oscillations were greatly reduced. The impact of I_h_ was not bigger when the external action potentials were delivered at the resonance frequency of the h-channels.

## Methods

To investigate the effect of I_h_ on amplitude fluctuations in ongoing oscillations, we built, as in Avella Gonzalez et al. (2012), a neuronal network consisting of 80 excitatory cells and 20 inhibitory cells, reflecting the ratio of excitatory to inhibitory cell numbers found in most cortical areas (Markram et al., 2004). The network was large enough to capture the network dynamics, yet small enough to be able to run many simulations of 40 s duration for different input conditions and ion-channel compositions (i.e., with and without h-channels). The network was implemented in the simulation environment NEURON (Hines and Carnevale, 1997), and the results were analyzed in MATLAB.

### Cells

Both excitatory and inhibitory cells consisted of a single compartment with a length and diameter of 20 μm. The membrane contained the Na^+^ and K^+^ channels responsible for action potential generation, as well as leakage channels and h-channels (Bender et al., 2005; Aponte et al., 2006). The leakage channels were modeled as a simple resistive component, whereas the h-channels and Na^+^ and K^+^ channels followed the Hodgkin-Huxley formalism (Hodgkin and Huxley, 1952a,b).

The change in membrane potential *V* (in mV) was given by

$$\begin{array}{l} C\frac{dV}{dt}=I_{\text{CDC}}-g_{\text{K}}n^{4}\left(V-E_{\text{K}}\right)-g_{\text{Na}}m^{3}h\left(V-E_{\text{Na}}\right)\\ \;-g_{\text{L}}\left(V-E_{\text{L}}\right)-g_{\text{h}}l\left(V-E_{\text{h}}\right)-g_{\text{GABA}}\left(V-E_{\text{GABA}}\right)\\ \;-g_{\text{AMPA}}\left(V-E_{\text{AMPA}}\right)-g_{\text{AP}}\left(V-E_{\text{AP}}\right) \end{array}$$

where *t* is time in ms; *C* = 10^−6^F/cm^2^ is the membrane capacitance; *g*_K_ = 800pS/μm^2^ and *E*_K_ = −100mV are the maximal conductance and reversal potential of the K^+^ channels; *g*_Na_ = 1000 pS/μm^2^ and *E*_Na_ = 50 mV are the maximal conductance and reversal potential of the Na^+^ channels; *g*_L_ = 1 pS/μm^2^ and *E*_L_ = −67 mV are the conductance and reversal potential of the leakage channels; and *g*_h_ = 5 pS/μm^2^ and *E*_h_ = −30 mV are the maximal conductance and reversal potential of the h-channels. Each cell could receive synaptic input from other cells in the network, with *g*_AMPA_ and *E*_AMPA_ the synaptic conductance and reversal potential of the excitatory AMPA channels; and *g*_GABA_ and *E*_GABA_ the synaptic conductance and reversal potential of the inhibitory GABA_A_ channels (for parameter values, see Section Network). In addition, each cell could receive two types of external input: a constant depolarizing current *I*_CDC_ and a train of external action potentials impinging onto an excitatory synapse, with synaptic conductance *g*_AP_ and reversal potential *E*_AP_ (for parameter values, see Section External Drive). Parameter values were based on Jensen et al. (2005) and Wang et al. (2004). Parameter values of the h-channels were obtained from Magee and Carruth (1999).

The dynamics of the gating variables *n, m*, and *h* (collectively denoted by *z*; note that *h* refers to a gating variable of the Na^+^ channel, not to a property of the h-channels) of the Na^+^ and K^+^ channels were given by

$$\begin{array}{l} \frac{dz}{dt}=\alpha_{z}\left(V\right)\left(1-z\right)-\beta_{z}\left(V\right)z \end{array}$$

where α_*z*_(*V*) and β_*z*_(*V*) are the voltage-dependent functions for the opening and closing rates. For the *n, m*, and *h* variables, these functions were (Wang et al., 2004; Jensen et al., 2005):

$$\begin{array}{lll} \alpha_{n}\left(V\right) & = & 0.032\left(V+52\right)∕\left(1-\exp\left(-0.2\left(V+52\right)\right)\right)\\ \beta_{n}\left(V\right) & = & 0.5\exp\left(-0.025\left(57+V\right)\right)\\ \alpha_{m}\left(V\right) & = & 0.32\left(54+V\right)∕\left(1-\exp\left(-0.25\left(V+54\right)\right)\right)\\ \beta_{m}\left(V\right) & = & 0.28\left(27+V\right)∕\left(\exp\left(0.2\left(V+27\right)\right)-1\right)\\ \alpha_{h}\left(V\right) & = & 0.128\left(\exp\left(-0.056\left(V+50\right)\right)\right)\\ \beta_{h}\left(V\right) & = & 4∕\left(1+\exp\left(-0.2\left(V+27\right)\right)\right) \end{array}$$

The dynamics of the gating variable *l* of the h-channels was given by (Magee, 1998; Migliore et al., 2004):

$$\begin{array}{l} \tau_{l}\left(V\right)\frac{dl}{dt}=l_{\infty }\left(V\right)-l \end{array}$$

where

$$\begin{array}{lll} \tau_{l}\left(V\right) & = & \frac{\exp\left(0.033\left(V+75\right)\right)}{0.02\left(1+\exp\left(0.083\left(V+75\right)\right)\right)}\\ l_{\infty }\left(V\right) & = & \frac{1}{1+\exp\left(\left(V+81\right)∕7\right)} \end{array}$$

Thus, τ_*l*_(*V*) has a bell-shaped form with a maximal value at *V* = −75 mV. The function *l*_∞_(*V*) has a sigmoid shape with a half-maximum of 0.5 at *V* = −81 mV. It approaches 0 for high *V* and 1 for low *V*; i.e., the channel is activated by hyperpolarized membrane potentials.

### Resonance

To verify that the model cell with h-channels exhibited resonance, we replaced the constant *I*_CDC_ by a sub-threshold ZAP current (i.e., a sine wave current whose frequency increases linearly with time) and determined the cell's transfer impedance for a broad range of frequencies, using the impedance tool from NEURON (Hutcheon et al., 1996), available at www.neuron.yale.edu/ftp/ted/neuron/izap.zip. The impedance is the complex ratio of the voltage to the current in an alternating current circuit; impedance thus extends the concept of resistance to situations with alternating currents. Aside from the ZAP current, the cell received no other input, thus no synaptic input and AP input. The ZAP current fluctuated in a sinusoidal way between 0.8 and 1.2 pA and was applied 100 ms after the start of the simulation; the whole simulation lasted 600 ms. The frequency of the ZAP current varied over time from 1 to 1000 Hz.

### Network

To build the network, we assigned to each cell a probability to connect to any other cell. As in Avella Gonzalez et al. (2012), excitatory (E) cells had a probability *P*_EI_ = 0.65 to connect to inhibitory (I) cells and a probability *P*_EE_ = 0.3 to connect to E cells. Likewise, I cells had a probability *P*_IE_ = 0.6 to connect to E cells and a probability *P*_II_ = 0.55 to connect to I cells. A connection consisted of a single synapse with a synaptic conductance as described below.

These connectivity values were chosen so that the network, in combination with the synaptic conductances (see below), generated oscillations through a PING (Pyramidal Interneuron Network Gamma) mechanism (Whittington et al., 2000). In this mechanism, which underlies most beta and gamma oscillations in the brain, the pyramidal cells (E cells) activate the interneurons (I cells), which in turn suppress the pyramidal cells. The PING mechanism requires strong connectivity from E to I cells, strong connectivity from I to E cells, and, to boost synchronous firing, connectivity among I cells (Whittington et al., 2000).

In the network, the E cells projected excitatory AMPA synapses onto E or I cells, and I cells made inhibitory GABA_A_ synapses onto E or I cells. The time course of synaptic conductance for both excitatory and inhibitory synapses was given by a mono-exponential function. The synaptic delay for both types of synapses was 1 ms (Bazhenov et al., 2008). The AMPA synapses had a conductance *g*_EE, EI_ = 1 pS/μm^2^, reversal potential *E*_AMPA_ = 0 mV and decay time constant τ_E_ = 2 ms (Börgers et al., 2005; Geisler et al., 2005; Bibbig et al., 2007). The GABA_A_ synapses had conductances *g*_II_ = 10 pS/μm^2^ and *g*_IE_ = 50 pS/μm^2^, reversal potential *E*_GABA_ = −80 mV and decay time constant τ_I_ = 10 ms (i.e., the IPSC decay time constant; Jensen et al., 2005). These parameters values were as in Bibbig et al. (2007) and Jensen et al. (2005) and resulted in rhythmic network activity (Whittington et al., 2000) with a frequency of about 18 Hz, within the frequency range reported for prefrontal cortex and CA1/CA3 hippocampal areas (Bibbig et al., 2007; van Aerde et al., 2008, 2009).

### External drive

As in Börgers et al. (2005), each cell could receive two kinds of external input: (i) a constant depolarizing current *I*_CDC_, representing cholinergic input required to induce the oscillations generated by the synaptic interactions between excitatory and inhibitory cells (Tiesinga et al., 2001; Widmer et al., 2006); and (ii) a train of external action potentials, representing background input from outside the network (Whittington et al., 1997; Börgers et al., 2005). As shown in our previous work (Avella Gonzalez et al., 2012), the minimal condition for producing alternating episodes of high and low oscillation amplitude is excitatory input from external trains of action potentials (APs) to I cells and a constant depolarizing current (CDC) to both E and I cells. This input protocol was also used here.

For each E and I cell, the amplitude of the current was randomly chosen from a uniform distribution, but fixed for the duration of a simulation. The amplitude was in the interval [10.1–11.3] pA for E cells and in the interval [3.8–6.3] pA for I cells. These values were based on Börgers et al. (2005) and Johansson et al. (1992).

Each I cell received a train of external APs impinging onto an excitatory synapse with conductance *g*_AP_ = 2.6 pS/μm^2^, reversal potential *E*_AP_ = 0 mV and decay time constant τ_AP_ = 2 ms. The train of external action potentials had a given randomness and mean firing rate. The randomness (AP-*rand*) was denoted by a number in the interval [0, 1], where 0 indicates no randomness and 1 indicates full randomness of the Poisson-distributed spike train. The mean firing rate (AP-*mfr*) was equal to 1/*isi*, where *isi* is the mean interspike interval. The first external spike occurred at *t* = *t*_on_; the firing times of all subsequent spikes were computed using

$$\begin{array}{l} t_{n+1}=t_{n}+\left(1-rand\right)\times isi+rand\times isi\times errand\left(\right) \end{array}$$

where *errand*() is a random number drawn from an exponential distribution in the interval [0,1]. The first spike was generated at *t*_on_ = 80 ms, and all cells received external trains of APs independently from each other.

### Analyzing network activity

As in Avella Gonzalez et al. (2012), network activity was analyzed separately for the excitatory and the inhibitory population. Results from the excitatory and the inhibitory population turned out to be very similar, so in most cases we report only about the excitatory population. To monitor the time domain of network activity, we constructed firing-rate histograms by counting spikes in time bins of 6 ms, which implies a sample frequency of about 167 Hz. A bin size of 6 ms practically excluded that more than one spike occurred per time bin per cell, so the number of spikes was generally equal to the number of firing cells per time bin.

To analyze the frequency domain of network activity, we performed a fast Fourier transform using the 6 ms-binned firing-rate histograms (of 40 s of activity). The histograms were first smoothened by convolving them with an alpha function *f*(*t*) = α^2^*te*^−α*t*^, where α = 0.27 and *t* is in 6 ms time units (the bin size); *f*(*t*) was evaluated for five consecutive time bins. The convolved signal was then used as input for the Welch's periodogram MATLAB algorithm.

To examine the time-frequency domain of network activity, we conducted a wavelet analysis on the convolved firing-rate histogram. We used the Torrence algorithm (Torrence and Compo, 1998) as implemented in MATLAB and a standard Morlet function with a frequency range of 0.01–70 Hz and 0.1 Hz scaling windows.

**Figure 1:**
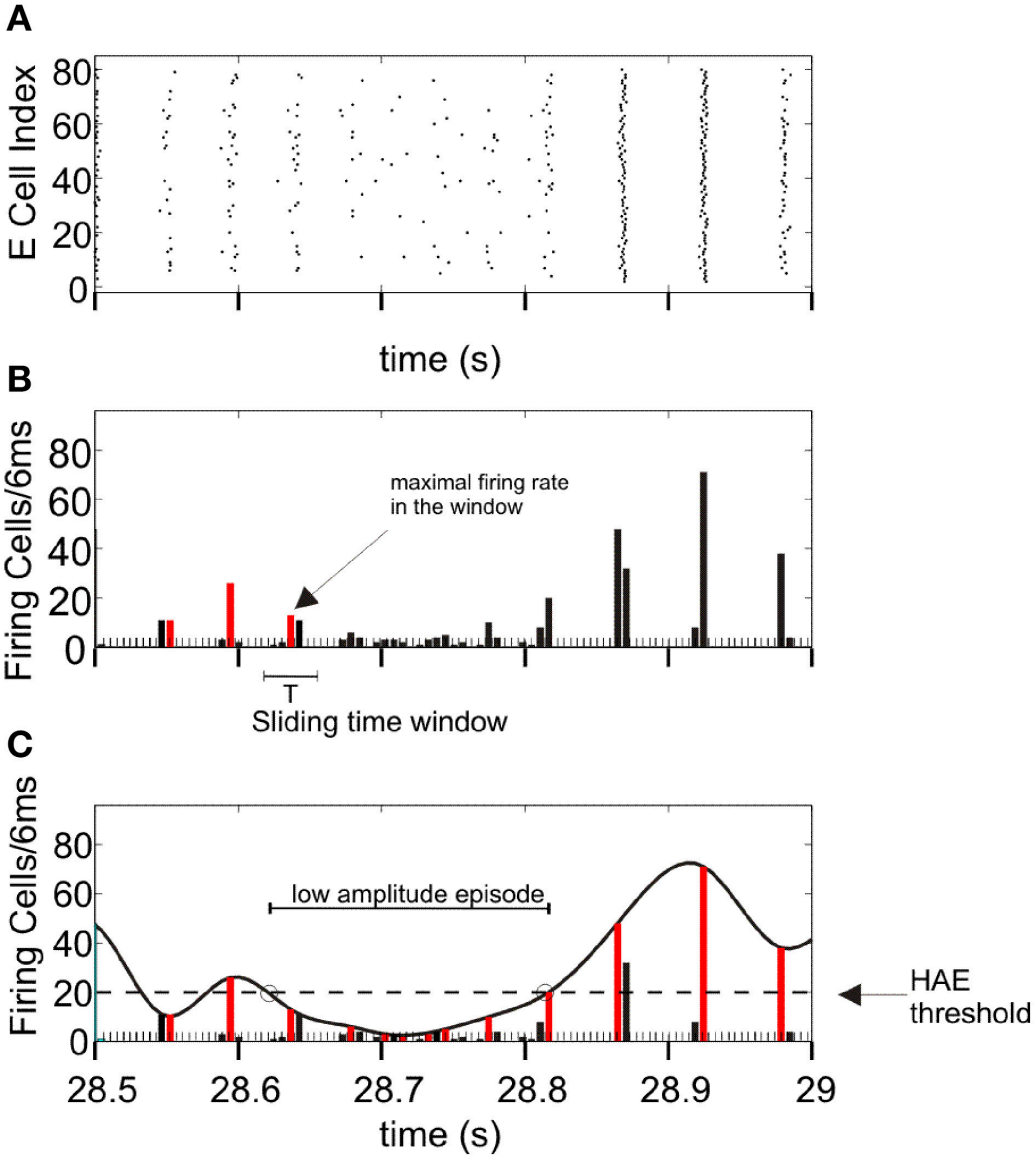
**Determination of high-amplitude episodes (HAEs) and low-amplitude episodes (LAEs) in network oscillations**. **(A)** Raster diagram showing the firing times (indicated by dots) of the excitatory cells. **(B)** Corresponding firing-rate histogram. The maximal firing rate (red bar) per oscillation period *T* is determined by using a sliding time window of length *T*. The time axis is divided into bins of 6 ms. **(C)** A spline polynomial is interpolated through the maximal firing rates (red bars). Time episodes during which the curve exceeds the HAE threshold (dashed line) are considered HAEs, otherwise LAEs (See further Section Analyzing Network Activity). From Avella Gonzalez et al. (2012).

To demarcate HAEs and LAEs, we used the same method as in Avella Gonzalez et al. (2012) (Figure 1). The method first determines the maximal firing rates in all successive periods of an oscillation, using a sliding time window of length *T*, where *T* is the oscillation period. The value of *T* is roughly estimated as the average time between the time bins in which the firing rate exceeds the mean firing rate. At the start, the first time bin *t*(1) with the highest firing rate is located, which marks the maximal firing rate in the first period of the oscillation. The sliding window is then centered around *t*(1), thus enclosing the range [*t*(1)*-T/*2*, t(*1*)+T/*2]. Next, the window is shifted to [*t*(1)+*T*/2, *t*(1)+*T*/2+*T*]. Within this range, time bin *t*(2) with the highest firing rate is searched for, which marks the maximal firing rate in the second period of the oscillation. Subsequently, the window is shifted to [*t*(2)+*T*/2, *t*(2)+*T*/2+*T*] to find time bin *t*(3), and so on. When the maximal firing rates in all periods are determined, a third-order spline polynomial is interpolated through the maximal firing rates. The interpolated curve is then used to delineate HAEs and LAEs. When during a particular time interval, the curve exceeds a given threshold, the interval is considered a HAE, otherwise a LAE. The HAE threshold is 0.25 × *n*_cells_, where *n*_cells_ is the total number of excitatory or inhibitory cells. Thus, a HAE is an episode in which at least 25% of the excitatory or inhibitory cells fired synchronously with a precision of 6 ms (the size of the time bins).

## Results

### I_h_ endows cell with resonance

Resonance is the property of a cell to respond selectively to inputs at a preferred frequency (Hutcheon and Yarom, 2000). To determine whether the model cell with h-channels exhibited resonance, we applied a sub-threshold ZAP current (i.e., a sine wave current whose frequency increases linearly with time) and determined the cell's transfer impedance for input frequencies ranging from 1 to 1000 Hz. The impedance is the complex ratio of the voltage to the current, so high impedance means a high voltage response. Apart from the ZAP current, fluctuating in a sinusoidal way between 0.8 and 1.2 pA, the cell received no other forms of input.

**Figure 2:**
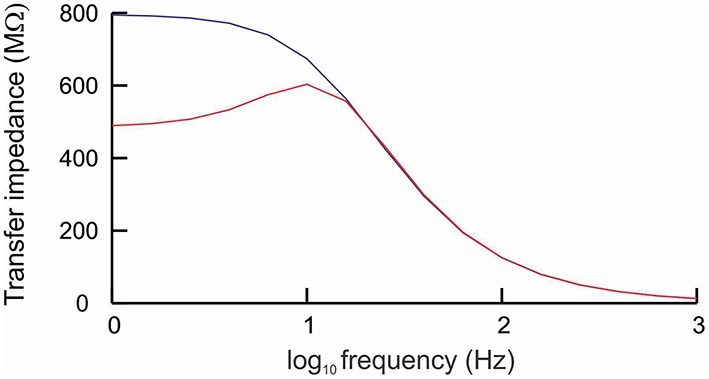
**Cell with h-channels behaves as a band-pass filter**. The cell was stimulated by a sine wave current whose frequency varied between 1 and 1000 Hz. In the absence of h-channels (blue line), the frequency response curve showed the typical low-pass filtering caused by the passive properties of the cell membrane. In the presence of h-channels (red line), the cell behaved as a band-pass filter, with elevated impedance in a window of about 6−15 Hz and a peak impedance at about 11.7 Hz (resonance frequency).

Without h-channels, the cell behaved as a typical low-pass filter (Figure 2) as a result of the cell's passive properties (Hutcheon and Yarom, 2000). The impedance was around 750 MΩ for frequencies below 10 Hz, and became increasingly lower for frequencies above this value. With h-channels, the cell acted also as a high-pass filter: for frequencies below 4 Hz, the impedance was markedly lower than without h-channels (Figure 2). The combination of low-pass and high-pass filtering resulted in elevated impedance in a window of about 6–15 Hz, with maximum impedance at 11.7 Hz (the resonance frequency).

The h-current (I_h_) is a slow, hyperpolarization-activated, depolarizing current that opposes changes in membrane potential (Chen et al., 2002; Biel et al., 2009; Kase and Imoto, 2012). As the cell hyperpolarizes, I_h_ activates, which decreases the hyperpolarization. Conversely, as the cell depolarizes, I_h_ deactivates, which attenuates the depolarization. In combination with the slow kinetics of I_h_, this ability of I_h_ to counter changes in membrane potential means that I_h_ acts as a high-pass filter. At low frequencies, the h-channels have time to open and to suppress potential changes, whereas at high frequencies there is not enough time and the potential changes remain unsuppressed. Together with the passive low-pass filtering caused by the membrane time constant, the presence of I_h_ thus endows the cell with resonance.

### I_h_ increases oscillation frequency

**Figure 3:**
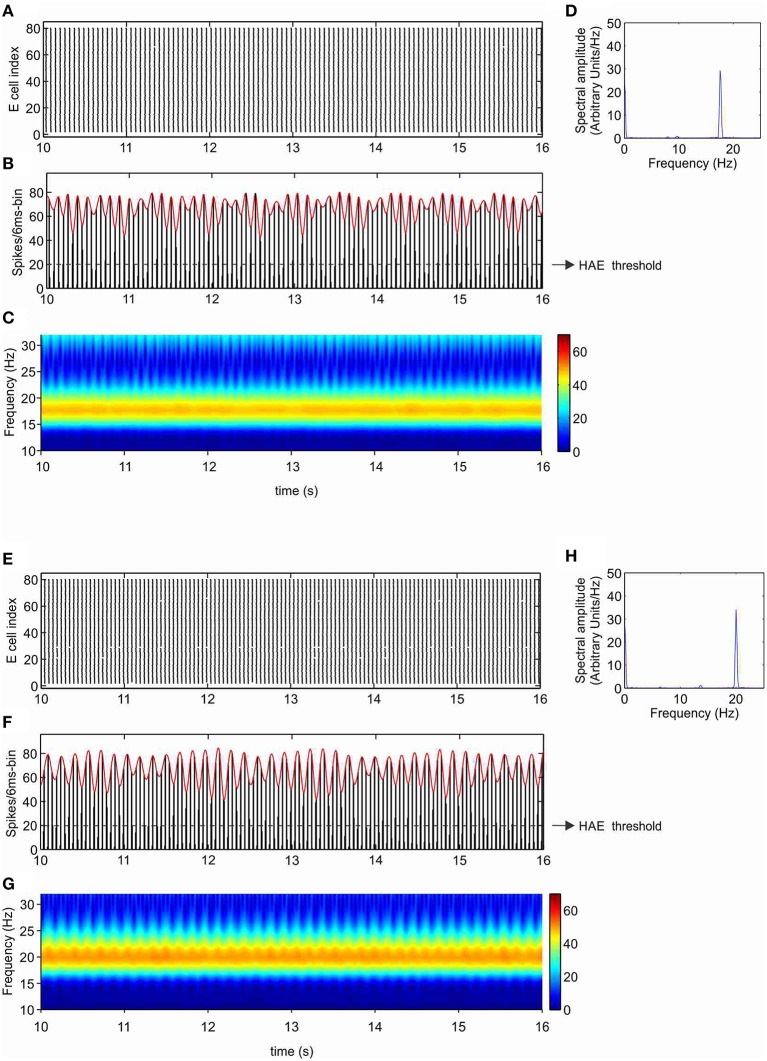
**I_h_ increases the oscillation frequency**. Shown are raster diagram of cell firing **(A,E)**, firing-rate histogram with interpolated spline polynomial (red line) **(B,F)**, wavelet transform **(C,G)**, and Fourier transform **(D,H)** of the excitatory population, in the absence **(A–D)** and presence **(E–H)** of h-channels in the network. The dashed line in **(B)** and **(F)** indicates the HAE threshold; time episodes during which the spline polynomial exceeds the threshold are considered high-amplitude episodes (in this case, the whole simulation period is a HAE). All cells received CDC input but no AP input, so there were no transitions to low-amplitude episodes (LAEs).

As shown in our previous work (Avella Gonzalez et al., 2012), the minimal stimulation condition for producing strong fluctuations in oscillation amplitude is a CDC to both excitatory (E) and inhibitory (I) cells and excitatory input from external trains of action potentials (APs) to the I cells. The CDC input represents cholinergic input, and the AP input reflects synaptic input from areas outside the network. The amplitude fluctuations arise from the interference between network-generated oscillations and AP input (Avella Gonzalez et al., 2012). Before examining how I_h_ affects amplitude fluctuations, we investigated the effect of I_h_ in a network that produced stable oscillations, without amplitude fluctuations. Figures 3A–D show a network without h-channels in which all cells received CDC input but no AP input. The network produced stable oscillations at a frequency of 17.8 Hz. The oscillations were caused by the interactions between the excitatory and inhibitory cells and were driven by the CDC input. In the presence of h-channels, neither the synchrony of cell firing nor the number of cells firing changed, only the oscillation frequency increased to 20 Hz (Figures 3E–H). The h-channels, which are partly open at rest, induce a depolarizing shift in the resting membrane potential, making it easier for action potentials to be triggered, which consequently leads to a higher firing rate and a higher oscillation frequency in the network.

### With I_h_, oscillation generation does not require external stimulation

**Figure 4:**
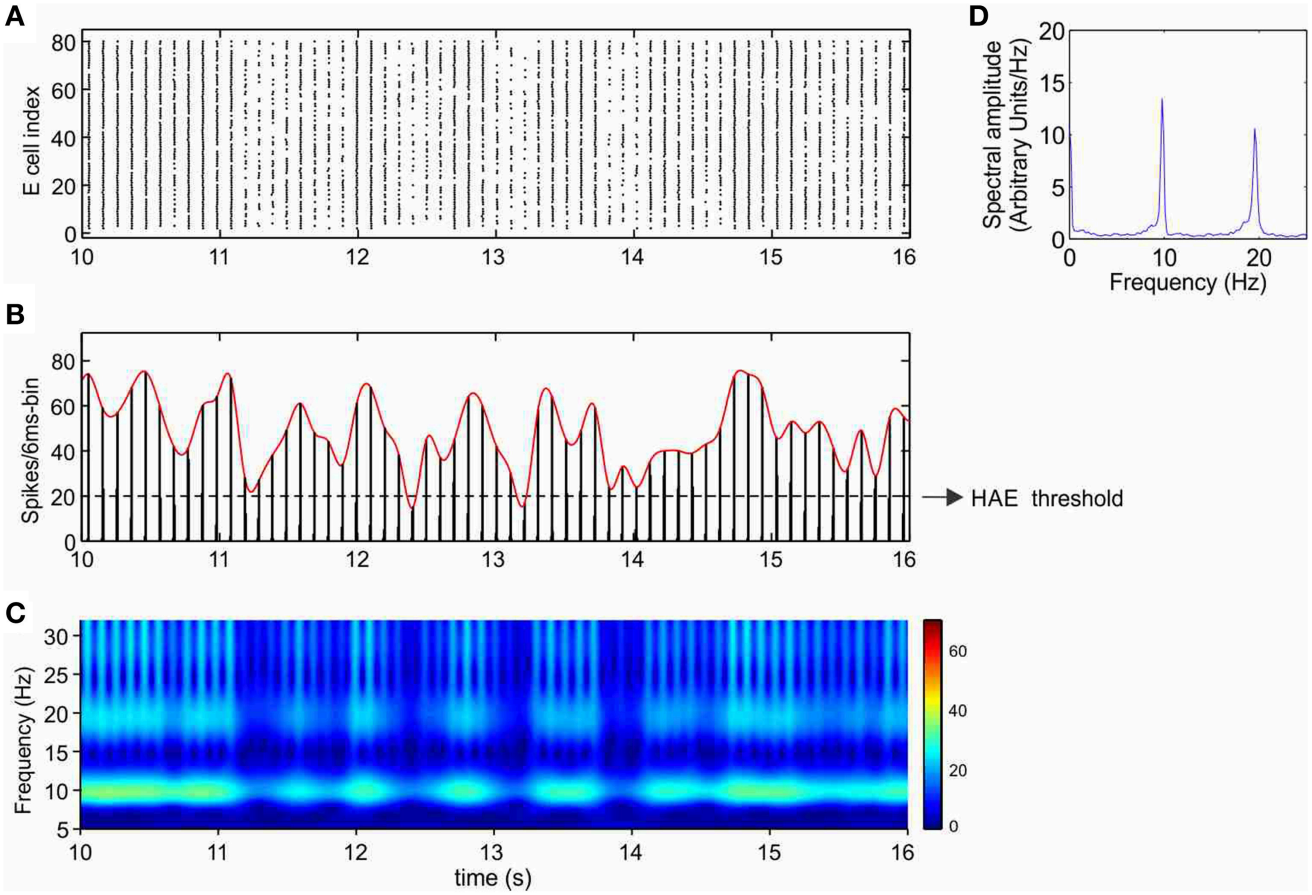
**In the presence of I_h_, oscillations are generated even without any form of external input (CDC, AP)**. Shown are raster diagram of cell firing **(A)**, firing-rate histogram with interpolated spline polynomial **(B)**, wavelet transform **(C)** and Fourier transform **(D)** of the excitatory population. The cells fired at a frequency of about 10 Hz. Note that owing to the highly synchronized activity, the Fourier transform **(D)** also produced a peak at a harmonic frequency (about 20 Hz), but there were no cells that actually fired at that frequency [see **(A)**]. There are large fluctuations in oscillation amplitude **(B)** that occasionally just drop below the HAE threshold. Cells had h-channels but did not receive CDC or AP input.

Without any form of external stimulation, i.e., CDC or AP input, the network without h-channels cannot generate oscillations and remains silent (Avella Gonzalez et al., 2012). Interestingly, in the presence of h-channels, the network did not need CDC or AP input to be able to produce oscillations (Figure 4). The oscillations, at a frequency of about 10 Hz, were more irregular than in the presence of CDC input (Figure 3), because not all cells participated in each oscillation cycle (Figure 4A), generating fluctuations in oscillation amplitude that occasionally just dropped below the HAE threshold (Figure 4B). Although not all cells fired in each oscillation cycle, the firing synchrony of cells that did fire was the same as in the presence of CDC input (compare Figure 3A and Figure 4A).

Cells with h-channels can display spontaneous firing because h-channels are partially open at resting membrane potential, supplying a depolarizing current that is responsible for starting an action potential. During the depolarizing phase of the action potential, the h-channels then slowly deactivate, but they become strongly activated again during the subsequent repolarization and after-hyperpolarization phase, triggering the next action potential.

### I_h_ strongly reduces HAE duration

**Figure 5:**
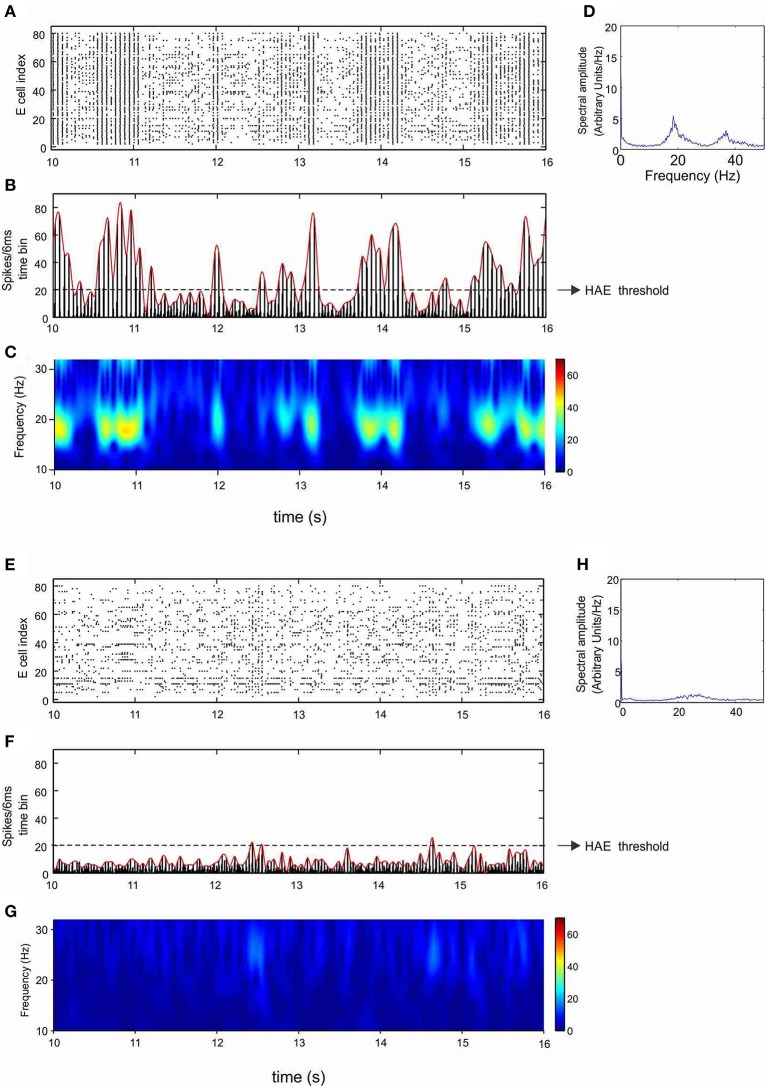
**I_h_ reduces the duration of high-amplitude episodes (HAEs)**. Shown are raster diagram of cell firing **(A,E)**, firing-rate histogram with interpolated spline polynomial **(B,F)**, wavelet transform **(C,G)**, and Fourier transform **(D,H)** of the excitatory population, in the absence **(A–D)** and presence **(E–H)** of h-channels in the network. Both in **(A–D)** and in **(E–H)**, all cells received CDC input, while the inhibitory cells received AP input with AP-*mfr* = 11.7 Hz and AP-*rand* = 1.

Next, we considered the effect of h-channels in a network that, in the absence of h-channels, produced irregular fluctuations in oscillation amplitude. As mentioned, the minimal stimulation condition for generating amplitude fluctuations is CDC input to both E and I cells and AP input to the I cells. Figures 5A–D show the excitatory population (results from the inhibitory population are very similar; see Supplementary Figure 1) in a network without h-channels that was stimulated using this minimal stimulation condition. HAEs are seen to alternate with LAEs (Figure 5B). During a HAE, cells fired highly synchronously, yielding high amplitudes in the firing-rate histograms and high power in the wavelet map (Figure 5C). During a LAE, the cells fired less synchronously (also fewer cells fired), as shown by the spreading out of activity over more time bins (see also Figure 1), leading to low amplitudes in the firing-rate histograms and low power in the wavelet map. The AP input disrupts the synchrony of firing, which reduces the oscillation amplitude, and a LAE commences (Avella Gonzalez et al., 2012). After some time, the E-I cell interactions drive the network back to synchrony, and a HAE begins. The tendency of the E and I cells to synchronize firing (Whittington et al., 2000) continually competes with the desynchronizing effect of the AP input, so HAEs constantly alternate with LAEs.

Figures 5E–H show the same network but then in the presence of h-channels. The h-channels disrupted the regular oscillations, with highly desynchronized firing as well as fewer cells firing, resulting in low amplitudes in the firing-rate histogram (Figure 5F) and low power in the wavelet map (Figure 5G). The dynamics of alternating HAEs and LAEs almost completely disappeared, and the network was in a LAE for almost the entire duration of the simulation. Although the power was very low, the peak frequency (27 Hz; Figure 5H) was higher than in the absence of h-channels (18 Hz; Figure 5D). Thus, in a network with HAE-LAE dynamics, the influence of I_h_ was much stronger than in a network that generated stable oscillations, in which introduction of h-channels affected only the oscillation frequency.

**Figure 6:**
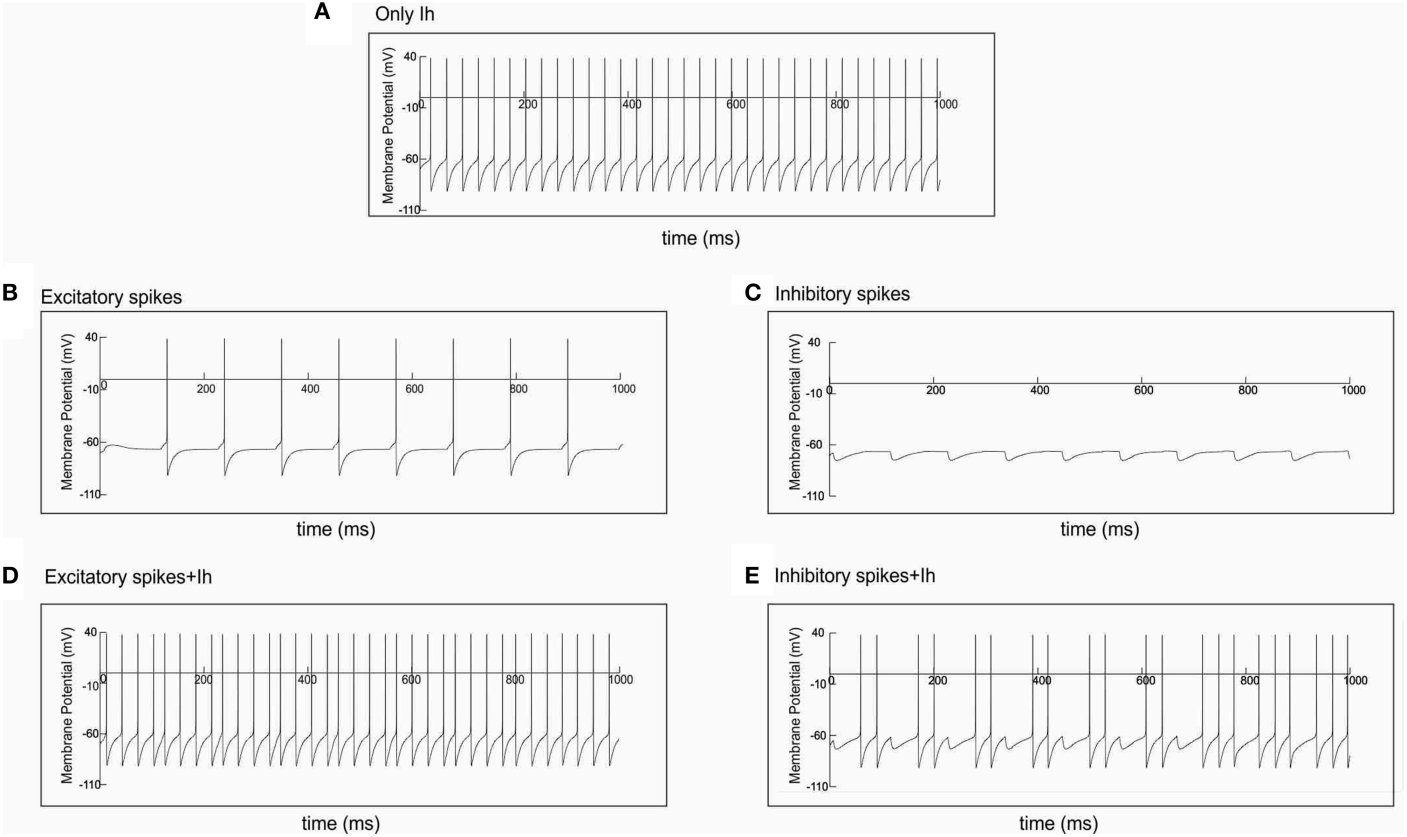
**Spike input disturbs cell firing in the presence of I_h_**. Single cell simulations illustrating the effect of action potential input, through an excitatory or an inhibitory synapse, on cell firing. **(A)** Without any form of input, the cell fired regularly in the presence of I_h_. **(B)** In the absence of I_h_, spike input through an excitatory synapse caused the cell to fire every time it received input. **(D)** In the presence of I_h_, the same spike input disturbed the regular firing shown in **(A)**, e.g., around 127 and 458 ms. **(C)** In the absence of I_h_, spike input through an inhibitory synapse caused the cell to hyperpolarize every time it received input. **(E)** In the presence of I_h_, the same spike input disturbed the regular firing shown in **(A)**. Firing was prevented, e.g., around 118 and 227 ms, or delayed, e.g., around 790 ms.

Because the h-channels are partially open at rest, they induce a depolarizing shift in the resting membrane potential, enabling the cells to fire even in the absence of input (see Section With I_h_, Oscillation Generation Does Not Require External Stimulation and Figures 4, 6A). Furthermore, the h-channels activate in response to hyperpolarization and deactivate in response to depolarization. As a result, the excitatory AP input onto the I cells (the condition for generating HAE-LAE dynamics) disturbs the firing of the I cells and also increases their firing rate (Figures 6B,D). In turn, the firing of the E cells (and other I cells), which receive inhibitory connections from the I cells, is prevented or delayed by the inhibitory input (Figures 6C,E), which strongly desynchronizes the network, leading to long LAEs (Figures 5E–G).

### I_h_ affects HAE duration for a wide range of AP input

**Figure 7:**
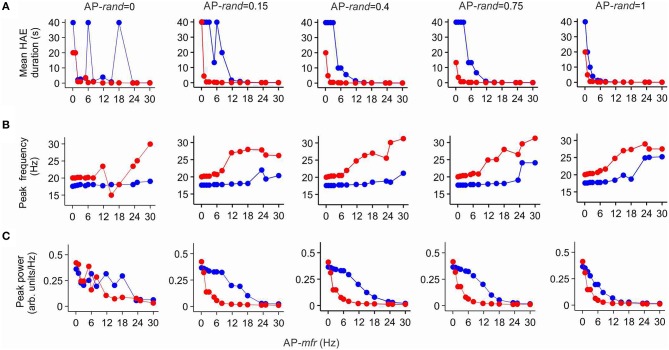
**I_h_ reduces the duration of high amplitude episodes (HAEs), increases the frequency, and decreases the power of network oscillations**. Shown are the mean HAE duration **(A)**, peak frequency **(B)**, and peak power **(C)** for different values of the randomness (AP-*rand*) and frequency (AP-*mfr* = 0.1, 1.0, 2.0, 3.0, 5.0, 6.0, 8.0, 11.7, 15.0, 18.0, 23.4, 25.0, 30.0 Hz) of the external action potential input (AP), both in the presence (red) and absence (blue) of h-channels in the network. The total simulation time was 40 s.

To investigate whether the randomness and frequency of the AP input affected its potential to disturb HAE-LAE dynamics and whether the AP input was more competent when it was delivered at the resonance frequency of the h-channels, we systematically varied AP randomness (AP-*rand* = 0, 0.15, 0.4, 0.75, 1.0) and AP frequency (AP-*mfr* = 0.1, 1.0, 2.0, 3.0, 5.0, 6.0, 8.0, 11.7, 15.0, 18.0, 23.4, 25.0, 30.0 Hz). Figure 7A shows the mean HAE duration as a function of AP-*mfr* for different values of AP-*rand*, both in the presence and in the absence of h-channels. Without h-channels, the mean HAE duration on the whole decreased (and the mean LAE consequently increased) with increasing AP-*mfr*, especially for AP-*rand* > 0.15. In general, a switch from a HAE to a LAE is more likely when the disruptive influence of the AP input is bigger (Avella Gonzalez et al., 2012). Thus, the higher the AP frequency and AP randomness, the shorter the mean HAE duration. With h-channels, the same trend was observed, but the mean HAE durations were much shorter than without h-channels. For AP-*rand* = 1, the difference between the HAE durations with and without h-channels was smaller than for the other values of AP-*rand* (e.g., compare AP-*rand* = 1.0 and AP-*rand* = 0.15 in Figure 7A). For most values of AP-*mfr*, the AP input desynchronized the oscillations and/or reduced the firing rate to such an extent that the mean HAE was very short (see close up of Figure 7A in Supplementary Figure 2). Only for the lowest values of AP-*mfr* (0.1, 1.0, 2.0 Hz) did substantial HAEs occur. Notice that, despite the presence of resonance in the sub-threshold domain (Section I_h_ Endows Cell with Resonance), the AP input did not have a bigger impact when it was delivered at the resonance frequency of the h-channels (11.7 Hz).

Figure 7B shows the peak frequency of the oscillations as a function of AP-*mfr* for different values of AP-*rand*, both in the presence and in the absence of h-channels. Without h-channels, the peak frequency slowly increased with increasing AP-*mfr*, but remained near 18 Hz for AP-*mfr* < 18 Hz. Around AP-*mfr* = 18–24 Hz, the oscillation frequency abruptly increased. Comparison with Figure 7A reveals that around this frequency, the HAE duration became very short, indicating a strong reduction in synchronous firing. For AP-*mfr* > 18–24 Hz, the network could not hold on to its own rhythm, and the firing frequency was more dictated by the external AP input than by the interactions between E and I cells. With h-channels, the peak frequency of the oscillations was higher than without h-channels. The peak frequency slowly increased with increasing AP-*mfr*, but stayed near 20 Hz for AP-*mfr* < 6 Hz. Around AP-*mfr* = 6 Hz, the oscillation frequency jumped to higher values. As in the case without h-channels, comparison with Figure 7A shows that around this value HAE duration became very short. For AP-*mfr* > 6 Hz, the oscillatory activity was strongly disrupted by the AP input, and the firing frequency in the network was determined more by the external AP input than by the E-I cell interactions.

Figure 7C shows the peak power of the oscillations as a function of AP-*mfr* for different values of AP-*rand*, both in the presence and in the absence of h-channels. For AP-*rand* > 0, the peak power was, except for AP-*mfr* = 0.1 Hz, systematically lower with h-channels than without h-channels. Thus, only when the disruptive influence of the AP input was relatively small, for AP-*mfr* = 0.1 Hz, did the presence of h-channels increase the power of the oscillations. In agreement with Figures 7A,B, the power quickly dropped to low values for AP-*mfr* > 18 Hz in the case without h-channels and for AP-*mfr* > 6 Hz in the case with h-channels. For AP-*mfr* > 18 Hz or AP-*mfr* > 6 Hz, fewer cells fired and/or they fired less synchronized, so the oscillation amplitude and thus the oscillation power greatly decreased (i.e., the network was in a LAE for most of the time). For AP-*rand* = 1, the difference between oscillation power with and without h-channels was smaller than for the other values of AP-*rand* (e.g., compare AP-*rand* = 1.0 and AP-*rand* = 0.15 in Figure 7C). As with the impact of AP input on HAE duration, notice that the AP input did not influence oscillation frequency (Figure 7B) or oscillation power (Figure 7C) more strongly when AP-*mfr* was at the resonance frequency of the h-channels (11.7 Hz).

### Impact of I_h_ diminishes with lower channel conductance

**Figure 8:**
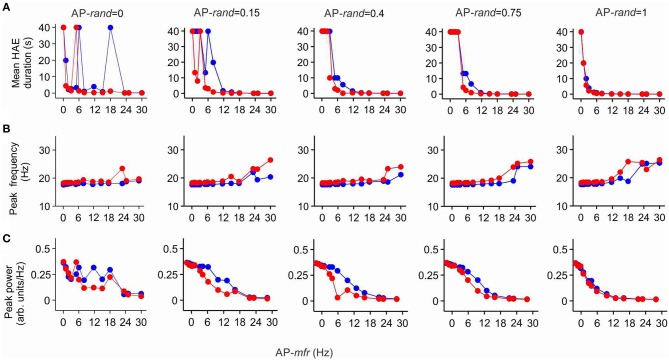
**The effect of I_h_ on high amplitude episodes (HAEs), frequency, and power of network oscillations diminishes when h-channel conductance is reduced**. The h-channel conductance was reduced to 25% of its default value. Shown are the mean HAE duration **(A)**, peak frequency **(B)**, and peak power **(C)** for different values of the randomness (AP-*rand*) and frequency (AP-*mfr* = 0.1, 1.0, 2.0, 3.0, 5.0, 6.0, 8.0, 11.7, 15.0, 18.0, 23.4, 25.0, 30.0 Hz) of the external action potential input (AP), both in the presence (red) and absence (blue) of h-channels in the network. The total simulation time was 40 s.

To examine whether lowering the maximal conductance of the h-channels would also diminish the impact of I_h_ on mean HAE duration, peak oscillation frequency and peak oscillation power, we reduced the maximal conductance to 25% of its default value. Figure 8 shows HAE duration, oscillation frequency and oscillation power as a function of AP-*mfr* for different values of AP-*rand*, both without h-channels and with reduced h-channel conductance (for close up of Figure 8A, see Supplementary Figure 3). As can be seen by comparing Figures 7, 8, the difference between presence and absence of h-channels was smaller with reduced h-channel conductance than with full h-channel conductance. This holds true for mean HAE duration, peak oscillation frequency and peak oscillation power. For AP-*rand* = 1, for instance, presence or absence of h-channels did hardly affect the results. When the h-channel conductance was reduced not to 25% but to only 50 or 75% of its default value, the influence of I_h_ was almost as strong as with full h-channel conductance (results not shown), indicating that I_h_ has a pronounced effect on oscillatory dynamics.

## Discussion

Using a computational model of a generic neuronal network, we here studied the impact of I_h_ on network oscillations generated by interacting excitatory and inhibitory cells. In particular, we looked at the influence of I_h_ on the occurrence of alternating high-amplitude (HAEs) and low-amplitude episodes (LAEs) in oscillations. In our previous work (Avella Gonzalez et al., 2012), we found that the minimal stimulation condition for obtaining HAE-LAE dynamics is a constant depolarizing current (CDC, representing cholinergic input) to both excitatory and inhibitory cells and excitatory input from trains of external action potentials (APs, reflecting synaptic input from areas outside the network) to the inhibitory cells. The HAE-LAE alternations arise because the AP input temporarily disrupts the synchrony of firing and so reduces the oscillation amplitude (Avella Gonzalez et al., 2012).

In networks lacking AP input, which produce stable oscillations without HAE-LAE alternations, insertion of h-channels increased the oscillation frequency, but had no effect on the synchrony of firing or the number of firing cells. In networks with AP input and HAE-LAE dynamics, I_h_, in addition to increasing the oscillation frequency, profoundly reduced the synchrony of firing and consequently decreased the oscillation amplitude, oscillation power and the mean HAE duration (and thus increased the mean LAE duration). The effect of I_h_ on HAE duration and oscillation frequency and power was not strongly dependent on the frequency (AP-*mfr*) or the randomness (AP-*rand*) of the external action potentials, and occurred for a wide range of AP-*mfr* and AP-*rand* values. Notably, the impact of I_h_ was not bigger when the AP input was delivered at the resonance frequency of the h-channels. The influence of I_h_ became less when the maximal conductance of the h-channels was lowered, although the conductance had to be reduced quite drastically before a diminished effect of I_h_ became noticeable.

Because h-channels are partially open at rest, I_h_ induces a depolarizing shift in the resting membrane potential, increasing the excitability of the cells and enabling them to fire even in the absence of input. Furthermore, I_h_ activates in response to hyperpolarization and deactivates in response to depolarization. As a result, networks with I_h_ are much more sensitive to the disruptive and desynchronising influence of the external excitatory AP input than networks without I_h_, leading to longer LAEs and shorter HAEs. As shown in our previous work (Avella Gonzalez et al., 2012), just adding a normal depolarizing current (e.g., a higher CDC input) instead of I_h_ yields longer rather than shorter HAEs, indicating that the special properties of I_h_ contribute to the impact of I_h_ on HAE duration. The effects of I_h_ on oscillation dynamics are not dependent on a particular choice of network parameters. A simulation with a different proportion of excitatory and inhibitory cells and different synaptic conductances yielded similar results (Supplementary Figure 4).

The predictions of the model could be tested experimentally in cortical slices cultured on multi-electrode arrays (MEAs). With MEAs, one can not only record field potentials but also deliver external electrical signals, so it could be tested whether HAE duration is reduced more by external input in the presence of I_h_ than in the absence of I_h_, when h-channels are blocked (Biel et al., 2009).

In a model of hippocampal CA3, with pyramidal, basket and oriens-lacunosum moleculare cells, Neymotin et al. (2013) also studied the impact of I_h_ on network rhythms. They found that I_h_ affected oscillation frequency and power, essentially through I_h_ influencing the firing rate of the inhibitory cell classes (basket and oriens-lacunosum moleculare cells). Unlike our study, they did not look at the effect of external input on synchronized firing (HAE-LAE dynamics) and the modulation of this effect by I_h_.

Synchronized firing between cells is important for correlation-based, Hebbian synaptic plasticity (Song et al., 2000). Since HAEs reflect episodes of enhanced firing synchrony, they provide favorable conditions for synaptic strength modification. The presence and maximal conductance of h-channels influence HAE duration and therefore may affect when learning and memory formation can take place.

Expression of h-channels differs between anatomical areas, with distinct neuronal circuits expressing subunits with different activation kinetics (Santoro and Tibbs, 1999). This differential expression may contribute to the different HAE and LAE distributions observed in different brain regions, e.g., the prelimbic and infralimbic regions of the prefrontal cortex (van Aerde, 2008; van Aerde et al., 2009; Avella Gonzalez et al., 2012). The expression of h-channels is not constant over time but undergoes long-term changes in response to altered network activity (Chen et al., 2002). In addition, neurotransmitter and neuromodulatory systems, such as those involving dopamine and acetylcholine, also influence I_h_(Chen et al., 2002). As our results suggest, this activity-dependent and –independent modulation of h-channels may affect oscillation frequency and amplitude fluctuations (HAE-LAE dynamics) and therefore could potentially influence all processes that depend on neuronal oscillations, such as synaptic plasticity, learning and memory, and attention.

Impaired expression of h-channels, with both up- and downregulation of I_h_ observed, is associated with the pathology of epilepsies (Chen et al., 2002; Biel et al., 2009). Upregulation of I_h_ was seen in hippocampal CA1 neurons, which, as in our model, was functionally coupled to an increased probability of action potential firing and a higher firing frequency (Chen et al., 2001). Downregulation of I_h_ was found in absence epilepsy (Ludwig et al., 2003), a type of epilepsy that is clinically defined by sudden, brief impairments of consciousness and behavioral arrest (absences). Downregulation of I_h_ was also found in temporal lobe epilepsy (Shah et al., 2004). Interestingly, in both absence and temporal lobe epilepsy, there is an increased prevalence of synchronous oscillatory activity in thalamocortical circuits (McCormick and Contreras, 2001) and entorhinal cortex (Shah et al., 2004), respectively, in line with our model result that I_h_, in combination with external input, desynchronizes network oscillations.

Various modulators of I_h_, both inhibitors and enhancers, have been proposed as anti-epileptic drugs (Chen et al., 2002). Our results indicate opposing effects of changing h-channel conductance with regard to controlling neuronal firing. Reducing I_h_ decreases firing and oscillation frequency but increases the synchrony of firing and thus increases oscillation amplitude and power. On the other hand, enhancing I_h_ increases firing and oscillation frequency but decreases the synchrony of firing and thus decreases oscillation amplitude and power.

### Conflict of interest statement

The authors declare that the research was conducted in the absence of any commercial or financial relationships that could be construed as a potential conflict of interest.
