# Supplementary material for: H-Channels Affect Frequency, Power and Amplitude Fluctuations of Neuronal Network Oscillations
Source: Front Comput Neurosci. 2015 Nov 19;9:141. doi: 10.3389/fncom.2015.00141 (PMC4652018; doi:10.3389/fncom.2015.00141)
Supplement: Supplementary file 1 [file DataSheet1.DOCX]

***Supplementary Material***

**H-channels affect frequency, power and amplitude fluctuations of neuronal network oscillations**

**Oscar J. Avella Gonzalez, Huibert D. Mansvelder, Jaap van Pelt and Arjen van Ooyen***

***Correspondence:** arjen.van.ooyen@gmail.com

**
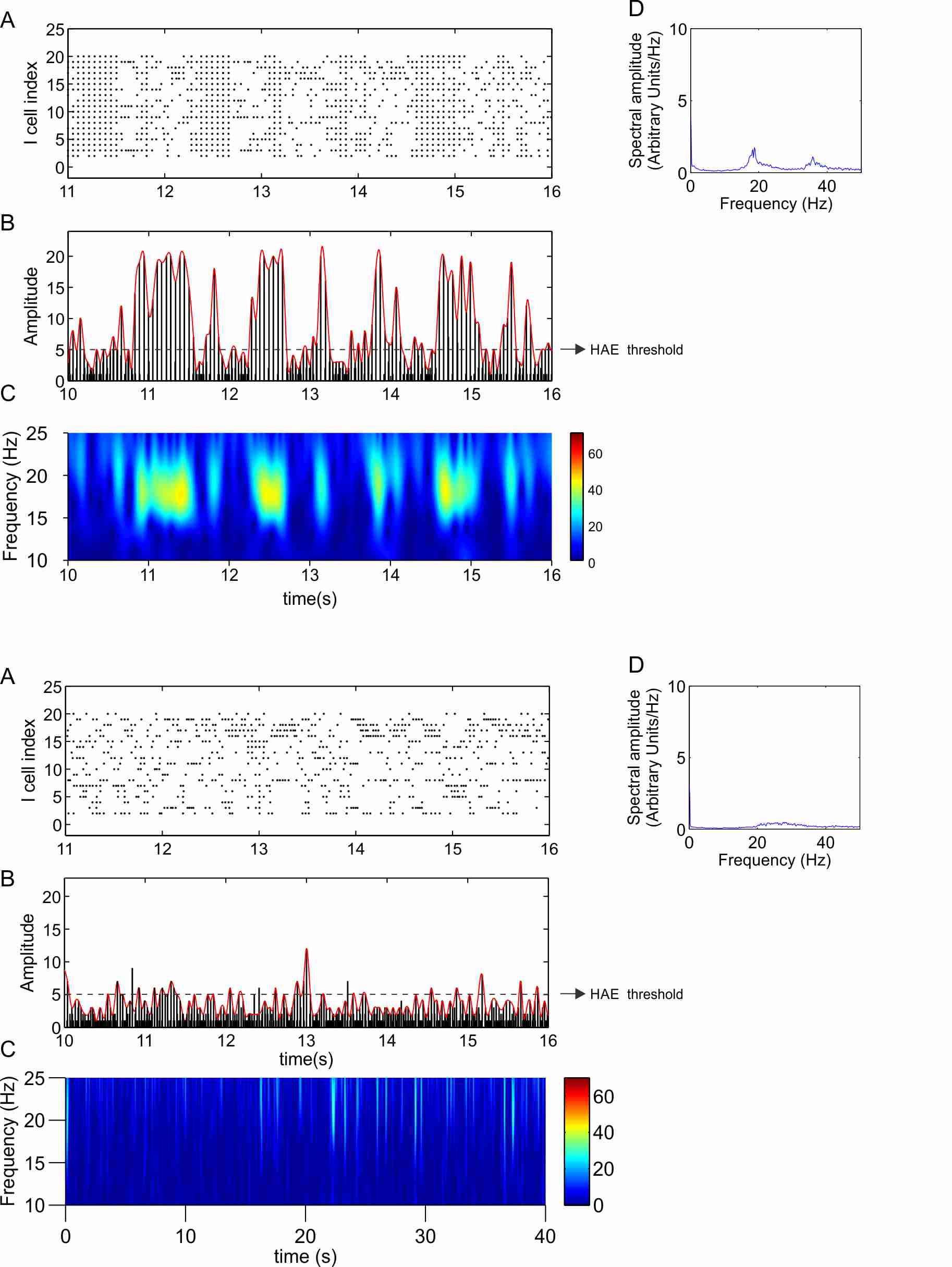
**

**Supplementary Figure 1.**  **I_h_ reduces the duration of high-amplitude episodes (HAEs).** Shown are raster diagram of cell firing **(A, E)**, firing-rate histogram with interpolated spline polynomial **(B, F)**, wavelet transform **(C, G)** and Fourier transform **(D, H)** of the inhibitory population, in the absence **(A-D)** and presence **(E-H)** of h-channels in the network. Both in **A-D** and in **E-H**, all cells received CDC input, while the inhibitory cells received AP input with AP-*mfr* = 11.7 Hz and AP-*rand* = 1.


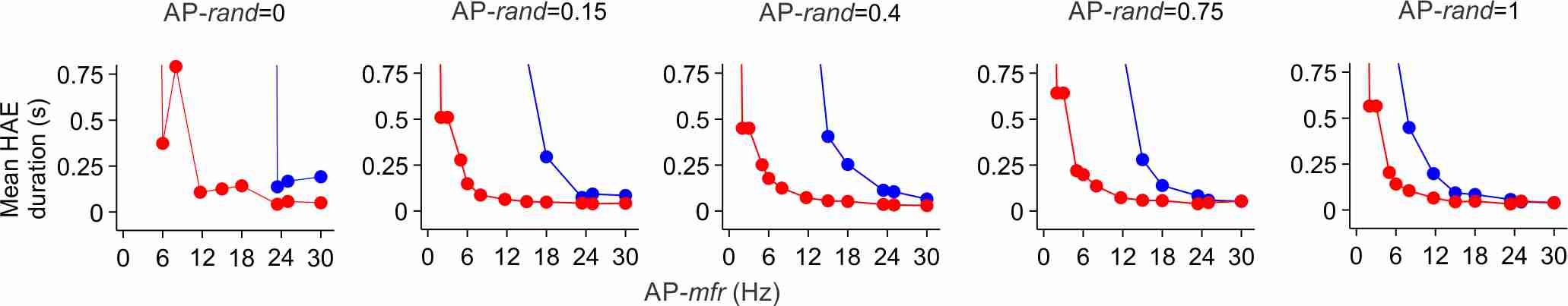


**Supplementary Figure 2. I_h_ reduces the duration of high amplitude episodes (HAEs).** The mean HAE duration for different values of the randomness (AP-*rand*) and frequency (AP-*mfr* = 0.1, 1.0, 2.0, 3.0, 5.0, 6.0, 8.0, 11.7, 15.0, 18.0, 23.4, 25.0, 30.0 Hz) of the external action potential input (AP), both in the presence (red) and absence (blue) of h-channels in the network. Close up of Fig. 7A, highlighting the short HAE durations for large AP-*mfr*.


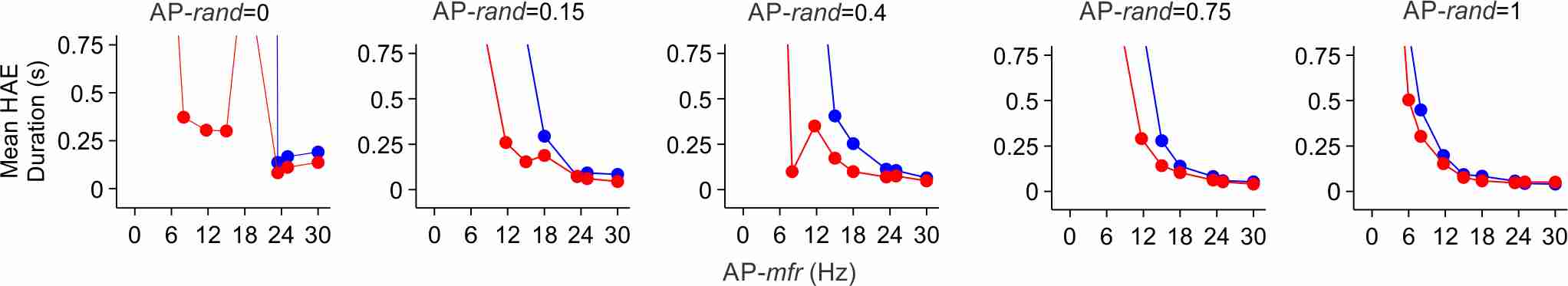


**Supplementary Figure 3. The effect of I_h_ on high amplitude episodes (HAEs) is reduced when h-channel conductance is 25% of its default value.** The mean HAE duration for different values of the randomness (AP-*rand*) and frequency (AP-*mfr* = 0.1, 1.0, 2.0, 3.0, 5.0, 6.0, 8.0, 11.7, 15.0, 18.0, 23.4, 25.0, 30.0 Hz) of the external action potential input (AP), both in the presence (red) and absence (blue) of h-channels in the network. Close up of Fig. 8A.


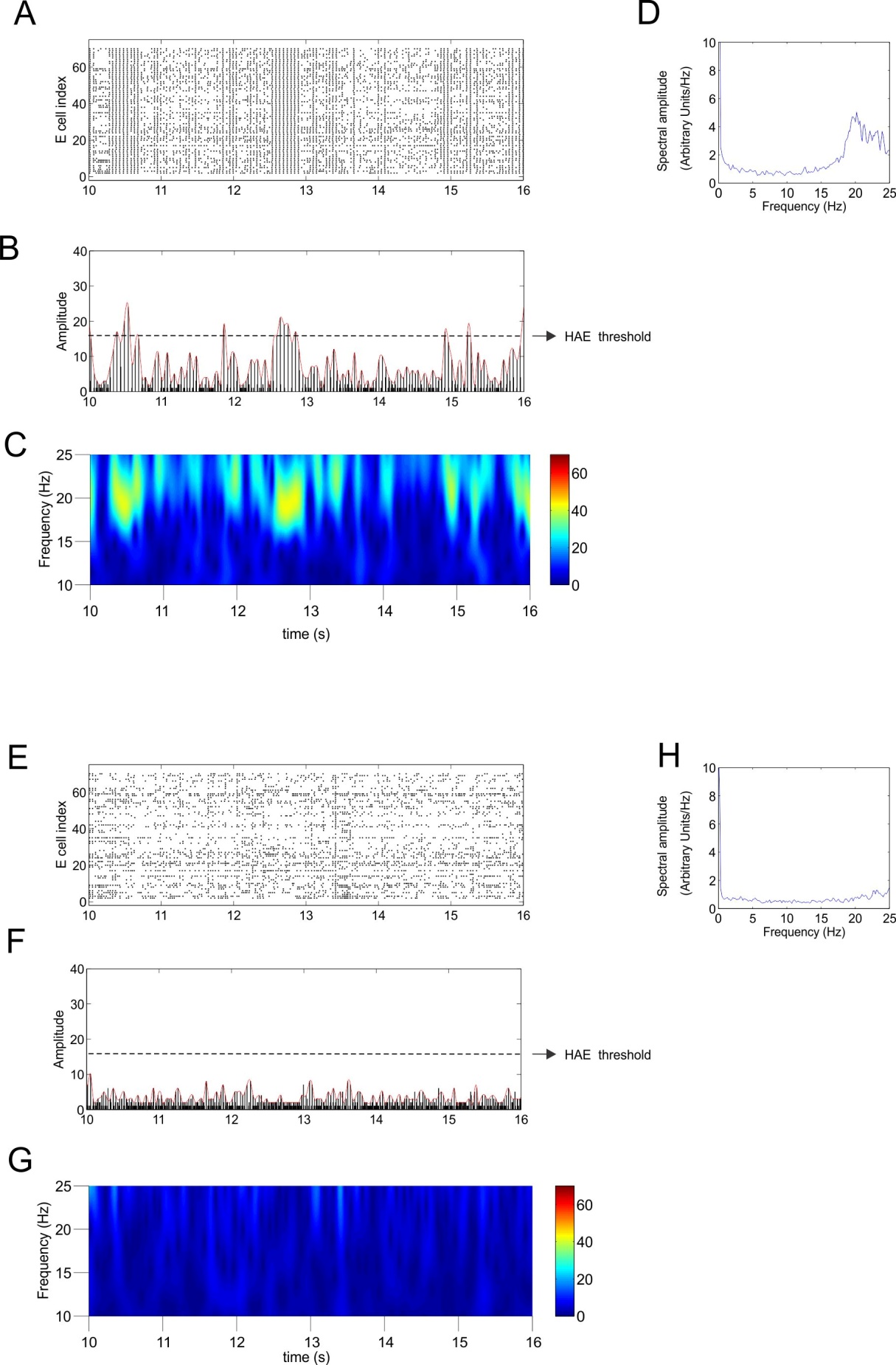


**Supplementary Figure 4. I_h_ reduces the duration of high-amplitude episodes (HAEs).** Shown are raster diagram of cell firing **(A, E)**, firing-rate histogram with interpolated spline polynomial **(B, F)**, wavelet transform **(C, G)** and Fourier transform **(D, H)** of the excitatory population, in the absence **(A-D)** and presence **(E-H)** of h-channels in the network. Both in **A-D** and in **E-H**, all cells received CDC input, while the inhibitory cells received AP input with AP-*mfr* = 11.7 Hz and AP-*rand* = 1. The network had 70 excitatory cells and 30 inhibitory cells. The synaptic conductance $g_{\text{EI}}$was 90% of its default value (see Section Methods), and the synaptic conductance $g_{\text{IE}}$was 60% of its default value. For the rest, all the parameter values were the same as in the default network.
